# Femtosecond Laser Densification of Hydrogels to Generate Customized Volume Diffractive Gratings

**DOI:** 10.1021/acsami.2c04589

**Published:** 2022-06-13

**Authors:** Zheng Xiong, Arun Poudel, Ameya R. Narkar, Zhe Zhang, Puskal Kunwar, James H. Henderson, Pranav Soman

**Affiliations:** † BioInspired Syracuse: Institute for Material and Living Systems, Syracuse University, Syracuse, New York 13244, United States; ‡ Department of Biomedical and Chemical Engineering, Syracuse University, Syracuse, New York 13244, United States

**Keywords:** femtosecond laser, densification, refractive index, hydrogel, diffractive grating, pH sensor

## Abstract

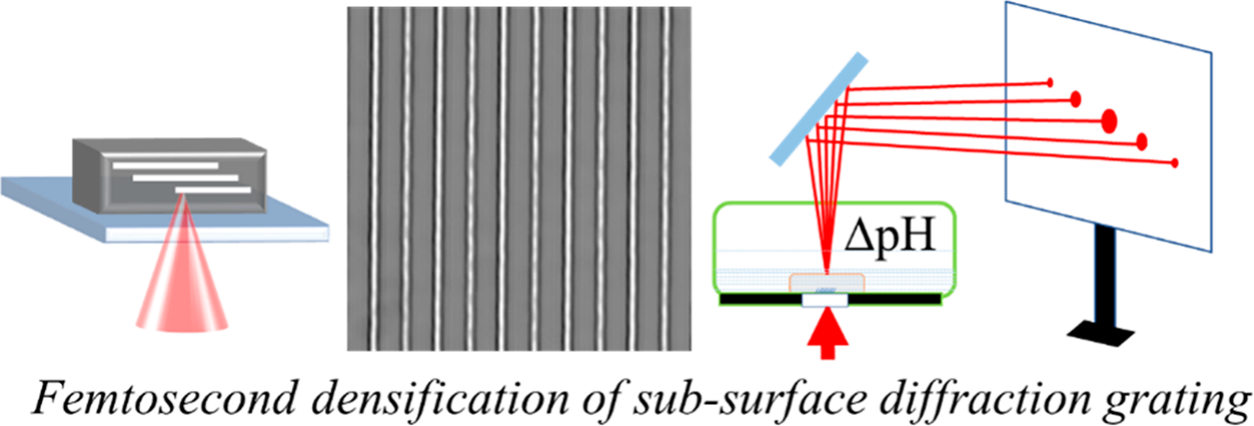

Inspired by nature’s ability to shape soft biological materials to exhibit a range of optical functionalities, we report femtosecond (fs) laser-induced densification as a new method to generate volume or subsurface diffractive gratings within ordinary hydrogel materials. We characterize the processing range in terms of fs laser power, speed, and penetration depths for achieving densification within poly(ethylene glycol) diacrylate (PEGDA) hydrogel and characterize the associated change in local refractive index (RI). The RI change facilitates the fabrication of custom volume gratings (parallel line, grid, square, and ring gratings) within PEGDA. To demonstrate this method’s broad applicability, fs laser densification was used to generate line gratings within the phenylboronic acid (PBA) hydrogel, which is known to be responsive to changes in pH. In the future, this technique can be used to convert ordinary hydrogels into multicomponent biophotonic systems.

## 1 Introduction

Evolution has provided many strategies to manipulate light by shaping ordinary materials (chitin, keratin, and cellulose) into hierarchical structures to satisfy specific biological functions, something even sophisticated man-made manufacturing finds challenging to replicate.^1^ For instance, many organisms are capable of changing the size or periodicity of nano-/microscale patterns to dynamically modulate their optical properties. Although such periodic patterns are commonly found on surfaces, organisms such as chameleons, cuttlefish, and squid have skin with subsurface or embedded patterns of crystals which, upon stimulation, modulate their periodicity, resulting in a change in skin color.^2−5^ Inspired by such natural photonic structures, researchers have developed new processing methods to generate custom patterns on or within ordinary materials and convert them into “smart” optical devices.^6,7^

Compared to conventional optical materials (semiconductors, glass, metals, and polymers), hydrogels have gained widespread utility in biomedical applications because of their close resemblance to the cellular microenvironment and their excellent optical transparency.^8−17^ Hydrogels exhibit 3D cross-linked networks of hydrophilic polymers, which allow diffusion of key analytes within their matrix.^18^ Methods based on lithography^19^ (E-beam,^20^ multibeam interference,^21^ nanoimprinting,^22^ and digital projection^15^) have been widely used to fabricate nano-/microstructures on hydrogel surfaces and modify their refractive indices (RI) and light shaping properties.^17^ However, for subsurface or volumetric processing of hydrogels in a user-defined manner, two-photon-absorption-based laser writing may be the only realistic method of choice as it can generate customized patterns within ordinary hydrogels with high design flexibility in 3D.^23−29^ Being embedded within a hydrogel matrix, printed structures are free from optical misalignment issues or damage during handling yet facilitate the modulation of pattern periodicity via analyte diffusion within the porous matrix. Previously, our group showed that femtosecond (fs) laser writing can generate “densified” structures within ordinary gelatin-based hydrogels, which can be used as biophysical cues to align cells encapsulated within the gelatin matrix.^30^ In the present work, we investigate whether fs laser densification induced RI change can be used to print custom volume diffractive gratings within ordinary poly(ethylene glycol) diacrylate (PEGDA) hydrogels and pH-responsive 3-(acrylamido)phenylboronic acid (PBA) hydrogels. We anticipate this design and processing strategy can be used to make integrated photonic systems using a large library of smart hydrogels.

## 2 Methods

### PEGDA Prepolymer Preparation

Poly(ethylene glycol) diacrylate (PEGDA, *M*_n_ = 700 Da) and phosphate buffered saline (PBS) were purchased from Sigma-Aldrich and used without further modification. The photoinitiator, lithium phenyl-2,4,6-trimethylbenzoylphosphinate (LAP), was synthesized by using a previously established protocol.^30^ The prepolymer solution was composed of varying amounts of PEGDA (10%–90%, v/v) with LAP (0.25%, w/v, for all compositions). The prepolymer solution was mixed for 10 min by using a magnetic stirrer, filtered (pore size = 0.2 μm), and used within 2 days after preparation. During the fabrication process, 30 μL of the prepolymer solution was pipetted onto a microscope glass slide (48 mm × 24 mm, Fisher Scientific) that was surface modified by using Sigmacote (Sigma-Aldrich). A PDMS spacer (Sigma-Aldrich) was placed between the microscope slide (bottom) and a glass coverslip (top) to control the prepolymer layer thickness of 1 mm.

### Characterization of PEGDA Prepolymer and Partially Cross-Linked PEGDA Samples

Refractive indices of cross-linked samples were directly measured by using digital refractometer (Sper Scientific). To characterize absorption properties, absorption spectra of the hydrogel samples held in plastic cuvettes were measured by using a scanning spectrophotometer from 250 to 1100 nm (Thermo Scientific). The elastic modulus was measured by using a standard rheometer (AR2000, TA Instruments, USA). Briefly, samples were prepared by casting the prepolymer solution in a PDMS mold (8 mm diameter, 100 μm thickness) and by partially cross-linking using UV light (power 3.5 mW cm^–2^, Omnicure S2000) for 10 s. Samples were then transferred to well plates and incubated in PBS for 24 h to facilitate removal of un-cross-linked prepolymer before testing them at 25 °C by using a rheometer (8 mm diameter bottom plate, gap = 0.5 mm). Storage modulus (*G*′) and loss modulus (*G*″) were measured at 0.5% strain for a range of 0.1–100 Hz. The elastic modulus was calculated by the equation *E* = 2*G*(1 + τ), where , where the Poisson’s ratio, τ, was assumed as 0.33. The linear regions of both moduli recorded between 1 and 10 Hz were used to calculate the elastic modulus (*E*). The swelling behavior was assessed by first immersing partially cross-linked hydrogel samples in PBS for 12 h under room temperature, followed by measuring the weight ratio between dried and swollen states with a laboratory balance (Thermo Scientific).

### Femtosecond Laser Writing Setup

**Figure 1:**
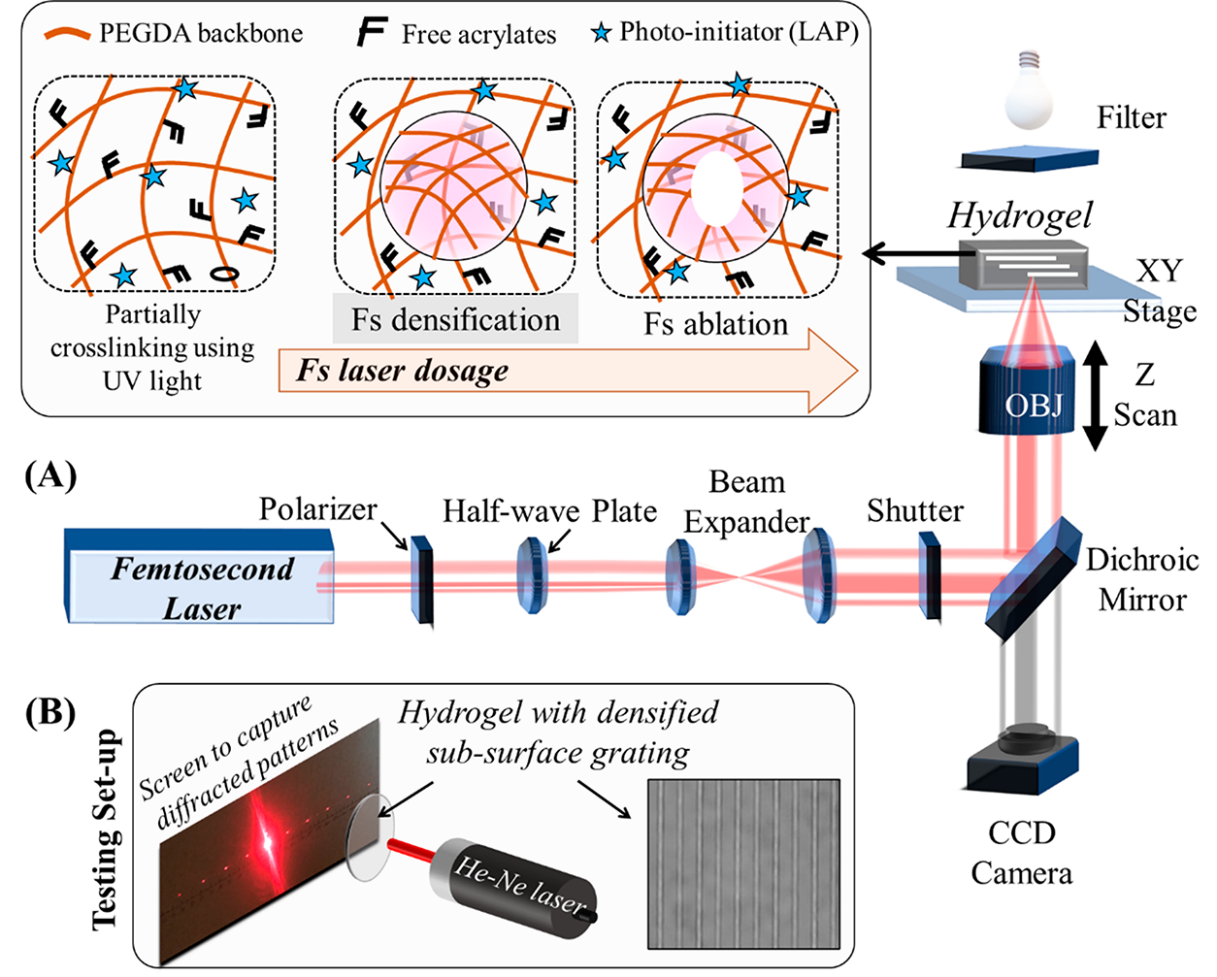
(A) Sketch of a custom-built femtosecond laser fabrication platform to generate subsurface densified or ablated patterns within the volume of partially cross-linked hydrogel. Inset depicts schematic of cross-linking of PEGDA monomers with exposure to a fs laser. With increased laser dosage, ablation of voids via cavitation is depicted. (B) Schematic of a testing setup to capture diffraction pattern generated by a hydrogel with embedded gratings.

A custom laser fabrication platform was designed and built by combining a wavelength-tunable Ti:sapphire fs laser (Coherent, Chameleon, USA) with a Zeiss microscope (Observer Z1, Germany), as shown in Figure 1A. The beam from the fs laser (wavelength tunable from 690 to 1080 nm, 80 MHz, 140 fs) is expanded 3 times to fully fill the back-aperture of an objective. The laser beam is directly focused inside a hydrogel sample, which is mounted on an *XYZ* stage of the Zeiss microscope. A λ/2 waveplate and Glan-Taylor polarizer (Thorlabs, USA) is combined to achieve a polarization-based power tuning system. A halogen bright light source (450 nm long-pass filter) is used for illumination, and a Hamamatsu FLASH4.0 V3 sCMOS digital camera is used to capture images of fs laser processing within hydrogels in real time. The whole system is automatically controlled by using the Visual Basic for Application (VBA) interface within the Zeiss microscope software.

### Femtosecond Laser Densification Process Flow

Hydrogel samples were fabricated in two steps. In the first step, the prepolymer solution composed of 90% PEGDA and 0.25% photoinitiator, LAP, was pipetted between two glass slides with a 1 mm thick PDMS spacer and then partially cross-linked. In the second step, the fs laser, focused by a high numerical aperture objective lens (NA = 0.55, 10×, Zeiss, Germany), was used to generate user-defined patterns by moving the stage controlled by a custom-written Visual Basic code (Figure 1). The laser dosage was changed by modulating the average power of the laser using the polarization-based tuning system or by modulating the scanning speed of the *XY* stage.

### Characterization of PEGDA Samples with Densified Subsurface Gratings

Post-writing, PEGDA samples were imaged by using phase-contrast (Lecia DM6000, Germany) and confocal microscopy (Zeiss observer X1). Samples were kept hydrated during phase-contrast microscopy whereas samples were incubated in Rhodamine B solution (10% w/v) for 2 min, washed three times with PBS before confocal imaging (10× objective). Images were reconstructed by using Zeiss Zen software. The setup shown in Figure 1B was used to visualize the diffraction patterns. Briefly, a He–Ne laser (11 mW, Thorlabs, USA) was used to irradiate samples with embedded gratings. A CMOS camera (Thorlabs, USA) placed 100 mm from the sample was used to capture the diffraction pattern. A power meter (Newport, USA) mounted on a linear stage (Thorlabs, USA) at the same location as the camera was used to measure intensity of each diffraction order.

### Fabrication and Characterization of Phenylboronic Acid (PBA) Hydrogel with Embedded Gratings

A precursor consisting of 1 M hydroxyethylacrylamide (HEAA), 40 mol % PBA, and 6 mol % methylenebisacrylamide (MBAA) (both PBA and MBAA were relative to HEAA) was dissolved in a solution containing 1585.5 μL of dimethyl sulfoxide (DMSO) and 2000 μL of deionized (DI) water. The photoinitiator, LAP, was maintained at 1 wt %. A 1 mm thick rectangular sample was prepared by pipetting the precursor solution within a space created by two standard glass slides and spacers. The whole construct was kept in a UV box (B9Creations Model Cure Light Box, λ = 390–410 nm, 65 *D*) and cross-linked for 4 s. This duration was chosen to keep the hydrogel in a partially cross-linked state. Samples were washed in DI water and soaked in 0.1% LAP solution for 10 min before fs laser densification of line gratings was performed at a power of 400 mW and a scanning speed of 400 μm/s with a 50× objective (0.55 NA, Zeiss). Resultant diffraction patterns were recorded by using a white screen placed 1.5 m from the sample. Samples were then exposed to solutions of varying pH, and changes in the distance between first-order maxima within the diffractive patterns were recorded. Briefly, Tris-HCl buffer solution (pH 8.59) was prepared, and the solutions of different pH value were obtained by adding a calculated amount of 1 M NaOH solution. The volume of NaOH solution required to reach a certain pH in 6 mL of buffer solution was recorded by using a pH meter (FiveEasy F20, Mettler Toledo), and during an experiment the same volume was added to the buffer solution to obtain the desired pH. Diffraction patterns were captured by a digital single lens reflex camera (Canon EOS Rebel T6i), analyzed by using ImageJ, and plotted in Origin. All experiments were repeated three times.

## 3 Results

### Characterization of PEGDA Prepolymer and Partially Cross-Linked Samples

**Figure 2:**
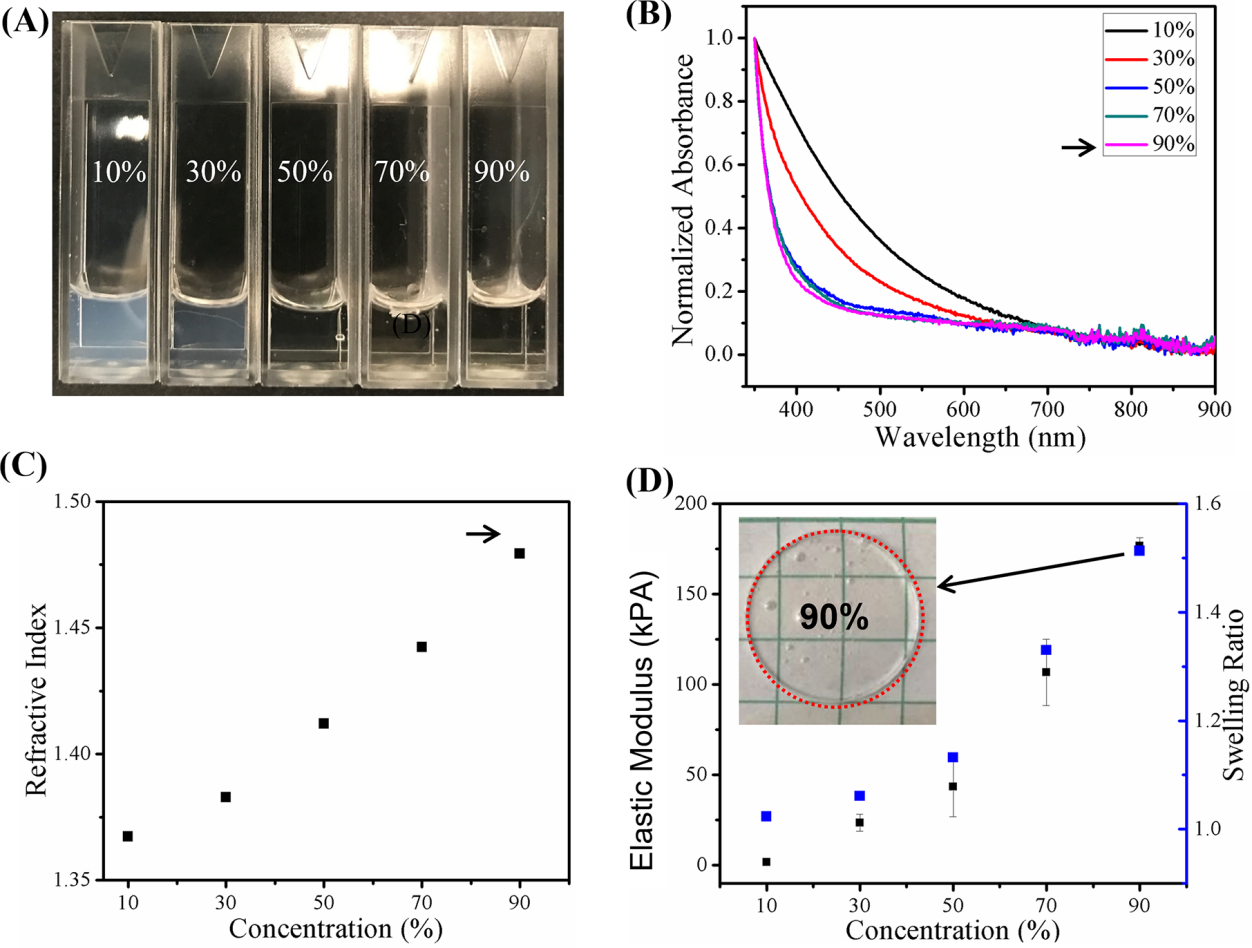
Characterization of PEGDA hydrogels with different concentrations (10%, 30%, 50%, 70%, and 90%). Gross visualization (A) and absorption spectrum plot (B) demonstrate that the optical transparency increases in visible wavelength range with an increase in PEGDA concentration. (C) Refractive index increases from 1.35 to 1.48 with increasing PEGDA concentration. (D) Elastic modulus and swelling ratio increase with an increase in PEGDA concentration. Black arrow in (B–D) point to the selection of PEGDA 90% as the base material for this work.

To develop durable samples, the PEGDA composition was optimized by using the dual criteria of high optical transparency and mechanical stability. We investigated the optical loss for PEGDA hydrogels by choosing a range of concentrations (10%, 30%, 50%, 70%, and 90% v/v) in Figure 2A. PEGDA hydrogels with 10% concentration held in a standard cuvette were white in color, indicating scattering across the visible spectrum. With increasing concentration, the PEGDA hydrogels became more transparent. (Figure 2B). PEGDA 10% had a normalized absorption of 0.4, while the absorption gradually dropped to 0.2 with increasing concentration over 50%. The refractive index (RI) increased linearly with the prepolymer concentration, reaching a maximum of 1.48 for 90% PEGDA hydrogels (Figure 2C). The elastic modulus of PEGDA hydrogels, as measured by rheometer, increased with increasing PEGDA concentration Figure 2D; for 90% PEGDA, the modulus was 176 kPa. Swelling tests were performed to investigate the stability of the optical properties in an aqueous environment. The swelling ratio in Figure 2D increased as PEGDA concentration increased from 1.05 to 1.51. For 90% PEGDA, circular sample showed no visible deformation or any changes in transparency (inset in Figure 2D). On the basis of these results, 90% PEGDA was chosen for all subsequent studies.

### Characterization of Densification Range as a Function of Laser Power, Speed, and Penetration Depth

**Figure 3:**
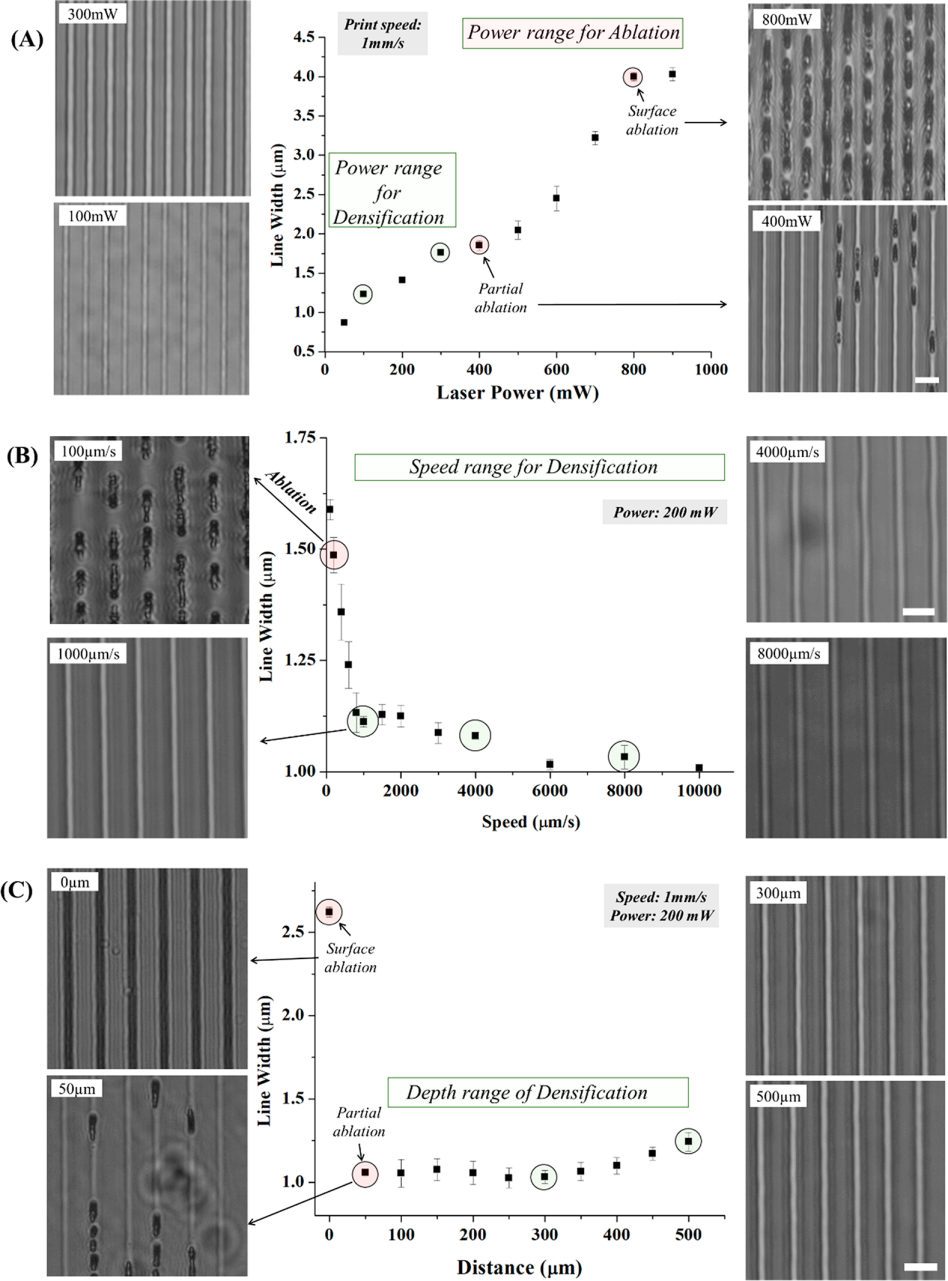
Characterization of fs laser hydrogel modification in the densification (green) and ablation (red) regimes as a function of laser power with constant print speed (A), processing speed with constant power (B), and processing depth with constant speed and power (C). Phase contrast images (top view) are shown for data points highlighted with a green circle (densification) or red circles (ablation). Scale bar: 10 μm.

Hydrogel samples (90% PEGDA) were partially cross-linked by using UV light, and these samples were used to generate defined embedded gratings via the fs laser densification process. The exposure time for UV cross-linking was carefully controlled to ensure the presence of free acrylate groups due to incomplete conversion of acrylate double bonds into covalent bonds. The fs laser dosage was increased by changing either the power or the stage speed based on the schematic of fs laser densification and ablation with increasing laser dosage (inset in Figure 1). At lower dosages, free acrylate groups are converted into covalent double bonds by two-photon cross-linking in the presence of photoinitiator (LAP).^31^ This process is different than regular single-photon UV cross-linking, as densification processing likely results in collapse and compaction of PEGDA chains, resulting in a region that appears brighter as compared with the rest of the UV-cross-linked PEGDA. Further increasing the laser dosage caused ablated cracks or voids. Next, we identified experimental conditions to achieve reliable densification of structures within 90% PEGDA. To do this, laser power, speed, and processing depth were systematically varied, and the resultant changes in the width of the line patterns were measured. An increase in laser power led to continuous material modification shifting from densification to ablation with a constant stage speed of 1000 μm/s. We also observed an increase in the width of the densified lines and the ablated microcracks (Figure 3A). Additionally, increasing the scanning speed resulted in a decrease in the width of densified lines with a constant power of 200 mW (Figure 3B). On the basis of these results, we chose a power of 200 mW and a stage speed of 1000 μm/s and characterized the processing depth range to reliably generate densified patterns. We also found that patterns could be generated in the range 100–500 μm below the surface (within the matrix) (Figure 3C).

### Calculation of Refractive Index Change Due to Densified Gratings

**Figure 4:**
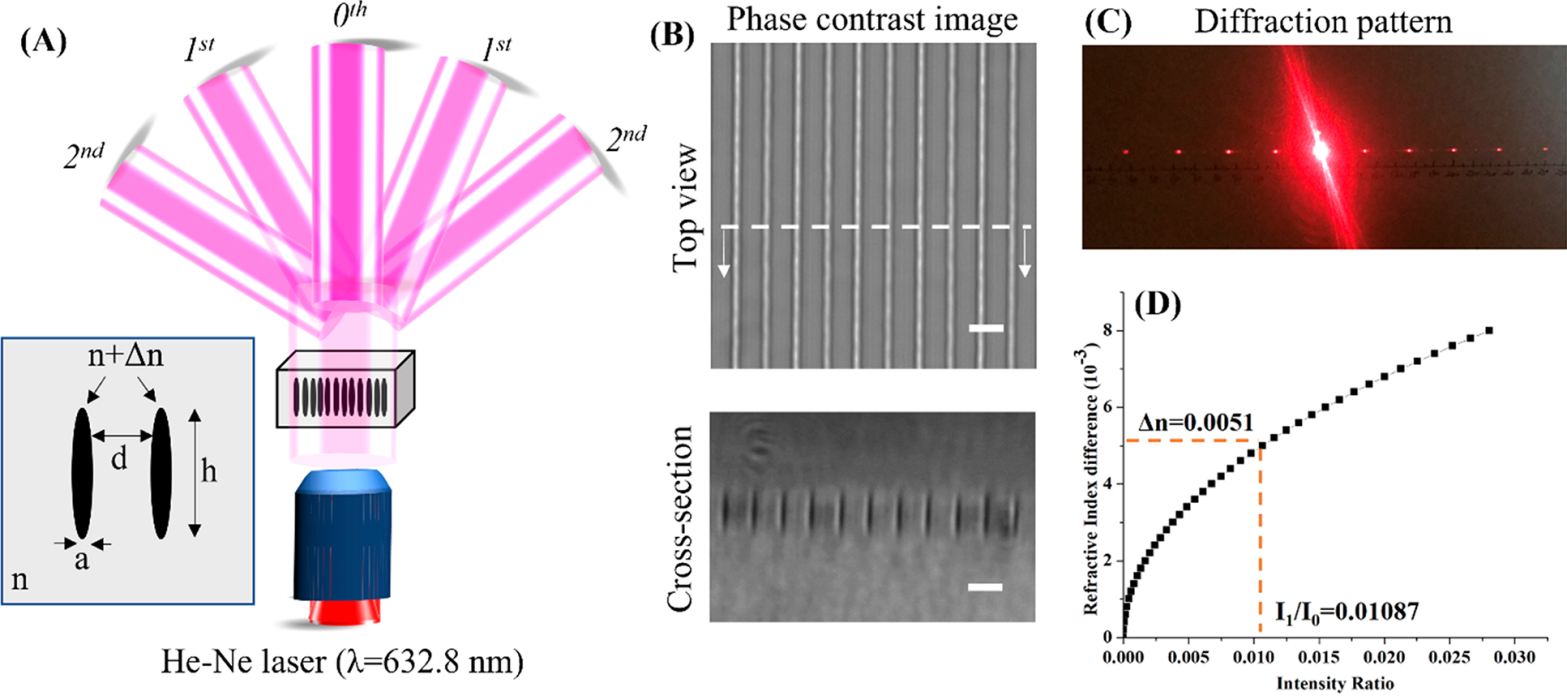
(A) Schematic of the testing setup with several parameters: *n* is the refractive index of base hydrogel while Δ*n* is the change in refractive index due to laser-induced densification of line grating structure with period, *d* = 10 μm, width, and *a* = 1 μm. (B) Phase contrast image (top view) of a densified line grating with a higher RI as compared to the surrounding materials. The cross section shows the grating height, *h* = 14.52 μm. (C) Photograph of the grating’s diffraction pattern. (D) Intensity ratio between zeroth diffraction order and first diffraction order and theoretical calculation of corresponding refractive index change (Δ*n* = 0.0051). Scale bar: 10 μm.

An optimized laser power of 200 mW and a scanning speed of 1000 μm/s were used to obtain microstructures within 90% PEGDA hydrogel samples. A schematic and a representative image of the fabricated phase grating at a depth of 200 μm within 90% PEGDA are shown in Figure 4A,B. Phase-contrast microscopy imaging (top view) shows fs laser densified regions as brighter than the surrounding hydrogel, indicating an increase in RI of densified structures. The size (*a* = 1 μm) and spacing (*d* = 10 μm) give the duty ratio ρ as 1:10. The height, *h*, of the grating was measured as 14.52 μm by imaging the cross section of the sample. The RI change between densified and unmodified PEGDA can be represented by *n* + Δ*n*, where *n* is the RI of unmodified PEGDA. To measure the change in RI, a He–Ne laser beam (λ = 632.8 nm, 11 mW) was used to irradiate the embedded grating, and the energy distribution of the diffraction orders was measured. The diffraction pattern (Figure 4C) was captured by using a CMOS camera behind the sample. A power meter was used to measure the intensity at the zeroth, first, and second diffraction order of the pattern, and the RI change Δ*n* was calculated by using the diffraction efficiency at various diffraction orders. The efficiency as a function of the RI change is derived based on Fourier optics as follows.^32^ For a phase grating, the transmission function can be written as
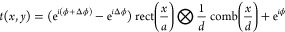
1where the original phase , the RI induced phase difference , *h* is the grating height, *d* is the period of the grating, and *a* is the line width. Here we assume the laser-induced RI change is uniform in the modified region. The diffraction spectrum *F*[*t*(*x*,*y*)] of eq 1 can be calculated by using Fourier transformation, and the actual diffraction energy distribution *I*(*x*,*y*) is the product of the diffraction spectrum conjugates:
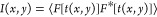
2On the basis of eq 2, the efficiency of *m*th diffraction order, η_*m*_, can be calculated by
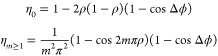
3where ρ is the duty ratio of the volume grating, in this case 1:10.

On the basis of eq 3, the theoretical relationship between the RI and the intensity ratio is plotted in Figure 4D. By taking the ratio of measured light intensities of diffraction orders first and zeroth (0.01087), the corresponding RI change within the densified grating is calculated as 0.0051. This result shows the potential of fs laser densification to make embedded diffraction components within hydrogels for biophotonics applications.

### Generation of Customized Subsurface Patterns

**Figure 5:**
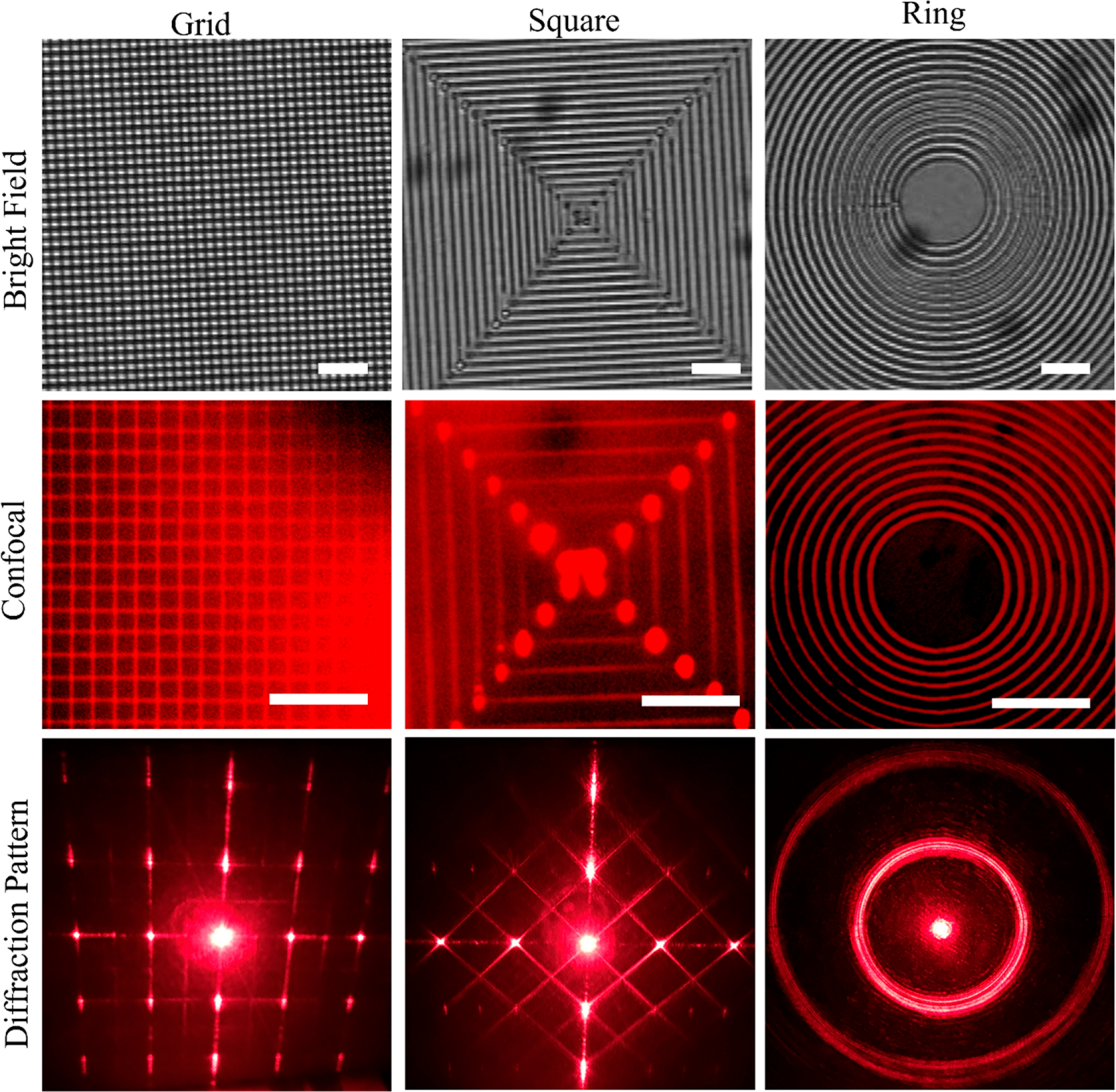
Custom-defined subsurface grid, square, and ring gratings generated by fs laser densification within partially cross-linked PEGDA hydrogel. Confocal images facilitated by Rhodamine B staining clearly highlight the increased density of embedded microstructures while photographs captured at far field show the generated diffracted patterns. Scale bar for bright field and confocal images: 50 μm.

Next, we used the optimized fabrication parameter (200 mW laser power and 1000 μm/s scanning speed) and generated several embedded beam shapers, including grid, square, and ring gratings, inside 90% PEGDA hydrogel by using the fs laser-induced densification method. Phase contrast and confocal images of densified patterns show higher brightness and fluorescence intensity respectively as compared to the surrounding material (Figure 5). Their optical properties of densified lines in terms of far-field diffraction patterns are also presented. Grid and square gratings exhibit symmetrical diffraction points, while the ring grating exhibits a central beam of high intensity (zeroth order) with associated concentric high-order cone beams. Minor distortion in some diffracted points can be attributed to misalignment of the He–Ne laser used for testing. This result shows that fs laser densification can be used to generate customized subsurface diffraction patterns within ordinary hydrogels.

### Densification within Phenylboronic Acid (PBA) Hydrogel

**Figure 6:**
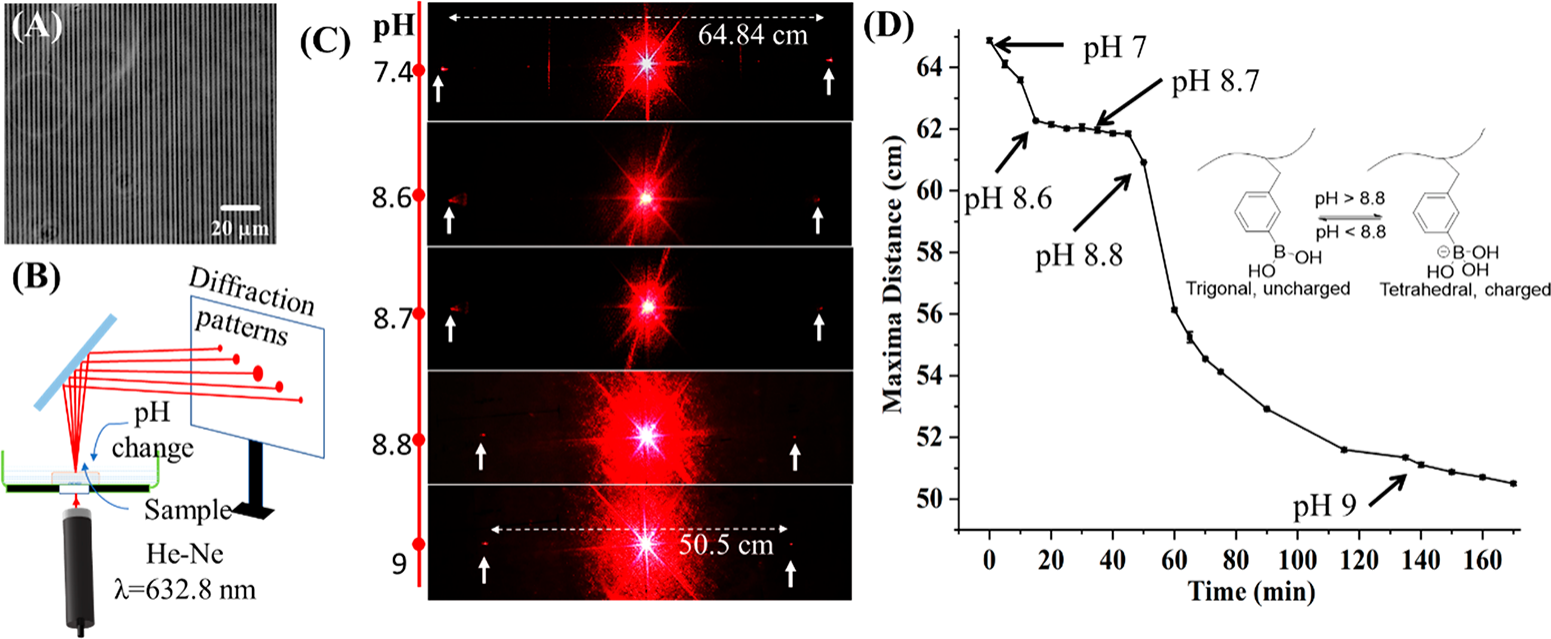
(A) Phase contrast image of subsurface grating densification within phenylboronic acid (PBA) hydrogel with periodicity of 3 μm. (B) Schematic of testing setup depicts capture of the diffraction pattern on a screen. (C) Series of photos showing the changes in the distances between first-order maxima when PBA hydrogels are immersed in a buffer solutions of different pH values. (D) Plot showing response dynamics of the PBA-based pH detector (marking shows the onset of pH detection).

Here, we tested whether fs laser densified line gratings generated in pH sensitive hydrogels can be used to detect pH by monitoring changes in grating periodicity. Partially cross-linked PBA hydrogel samples (UV light, 4 s) were used to pattern subsurface line gratings by using fs laser densification (Figure 6A). Upon exposure to solutions of varying pH, the hydrogels swelled, resulting in a change in the grating period, which in turn resulted in a change in the distances between first order maxima of the projected diffractive patterns (Figure 6C). The change in grating period (*d*) resulted in a change in the diffraction angle and changes in the first-order maxima spacing following the equation *g*_*m*_ = *L* tan ϕ_*m*_, where *g*_*m*_ is the distance between zeroth order with *m* order (in our case *m* = 1), *L* is the distance from sample to capturing screen (1.5 m), diffraction angle of *m*th order ϕ_*m*_ = sin^–1^(*k*λ/*d*) where *k* is integral number, λ is the HeNe wavelength (0.6328 μm), and *d* is the period of the grating (3 μm). Only lateral swelling of densified patterns was used for this characterization, as vertical swelling, if any, would be negligible due to the large distance between the sample and the capturing screen. Maximum swelling was observed beyond pH 8.8, with the swelling nearly plateauing beyond pH 9 (Figure 6D). Moreover, we observed a consequent decrease in the distance between two first-order maxima. Although this result shows that fs laser densification can be extended to other responsive hydrogels, more work needs to be done to improve the linearity, specificity, and accuracy of hydrogel detectors before they can be implemented in practice.

## 4 Discussion

Inspired by nature’s manufacturing strategy to create structural photonics, many strategies have been used to make hydrogel-based photonic devices for a range of biomedical applications.^17^ Most current methods focus on patterning the surface of natural and synthetic hydrogels (silk, chitin, chitosan, BSA, polyacrylamide, poly(acrylic acid) , PEGDA, and PDMS) for many photonic applications. However, fabrication of subsurface or volumetric gratings within hydrogels remains challenging. Other than fs laser densification, subsurface patterning can be also achieved by Multibeam interference lithography (MBIL) and bottom-up self-assembly-based methods; however, these methods have key limitations.^33,34^ For instance, MBIL can only fabricate periodic structures with geometries that are allowed by the incident angles, wavelengths, and intensities of the interfering beams. On the other hand, bottom-up strategies, although faster and scalable, rely on self-assembly processes optimized for specific materials and process conditions, limiting their use to fabricate custom-designed defect-free subsurface structures with precise orientation and/or alignment. Therefore, conversion of ordinary hydrogels into photonic elements with custom pattern designs remains difficult with these methods. In this work, we show that fs laser densification allows the transformation of ordinary hydrogels into photonic diffractive elements by fabricating user-defined subsurface grating structures.

As compared to conventional photonic materials, fs laser modification within hydrogels is still in its infancy. Previous work has shown that focusing ultrafast laser pulses inside transparent hydrogels induces localized permanent structural densification. Our results show that a RI change of 0.0051 between densified and surrounding hydrogel is sufficient to modulate the properties of light. This falls within the reported RI change range for polymeric materials.^34,35^ With increasing fs laser dosage, two regimes of densification and ablation were characterized. Below specific laser power, stage speed, and penetration depth thresholds (Figure 3), local densification was observed, while working above the thresholds resulted in the formation of hollow voids or unstable bubbles within the hydrogel matrix. In this work, we focus on the densification phenomenon.

At present, fs laser-induced densification in both conventional optical materials and hydrogels remains poorly understood because of the involvement of multiple nonlinear processes. Control experiments were performed to show that densification by a fs laser is distinct from simple photo-cross-linking using single-photon polymerization by conventional light sources (UV lamps and lasers). For instance, partially cross-linked samples with fs laser densified lines were irradiated with a UV lamp for up to 10 min to ensure complete cross-linking, and yet densified lines remain visible and appear brighter as compared to the surrounding hydrogel. We also found that the presence of photoinitiator (LAP) is necessary for fs laser densification. Others have reported no change in Raman spectra of densified lines, an indicator of material chemistry, even with a significant RI change of 0.06.^25,27,29,36^ This suggests that densification-induced RI change below the damage threshold of the hydrogel does not change the polymer composition, which implies additional cross-linking of the same hydrogel material due to local heat accumulation at the laser focus. The underlying cause of RI change via fs laser densification within hydrogels remains unclear, but it likely involves collapse or entanglement of polymer chains or water expulsion from densified regions due to local heat accumulation at the laser’s focal point.

Surface patterning of nano-/microstructures on hydrogels that respond to specific analytes (pH, glucose) by swelling is an active research field. PBA-based hydrogels have been used to detect analytes such as glucose,^37^ CO_2_,^38^ temperature,^39^ and pH.^40^ In this work, solutions of varying pH were used to characterize changes in diffractive patterns of embedded line grating. Subsurface densified patterns show a narrow range of detection with a nonlinear response. The nonlinearity in the pH plot is linked to the p*K*_a_ value of PBA ∼ 8.8^41^ (Figure 6D). At pH < p*K*_a_, the hydrogel sensor is in its equilibrium state because the PBA exists in an uncharged trigonal conformation. As the hydrogel approached the p*K*_a_ value of phenylboronic acid (∼8.8), this equilibrium shifts toward a charged tetrahedral structure, thus causing swelling of the hydrogel sensor and initiating a change in diffractive pattern spacing.

Before this method can be used to make hydrogel-based photonic devices, substantial work must be performed. For instance, the depth of subsurface patterning is limited by the transparency of the hydrogel matrix, which limits the method’s usefulness for making multilayered volumetric photonic elements. To resolve this issue, hybrid fs laser processing, a new method that is independent of materials’ optical properties, can be used to pattern densified structure at virtually any depth within the hydrogel matrix.^42^ The RI can also be enhanced by performing fs laser densification in the presence of dyes, nanoparticles, and metallic ions. Moreover, material properties and pattern designs need to be optimized to increase detection range, sensitivity, and specificity for biosensing applications.^43−46^ Overall, fs laser densification will pave the way for miniaturized and integrated hydrogel-based photonic systems where multiple photonic elements are embedded within a hydrogel matrix, a capability not achieved by surface structuring methods.^47^

## 5 Conclusion

We report a fs laser-induced densification method to change the effective RI within commonly used hydrogels. Optimized processing ranges for hydrogel densification were identified. Diffraction modeling and far-field diffraction measurements quantified densification-induced changes in the RI of 0.0051 in 90% PEGDA hydrogels. This effective RI change was used to pattern user-defined embedded gratings (line, grid, square, and ring) within PEGDA by using fs laser writing. This method was also extended to the responsive hydrogel PBA, and changes in pH were monitored by tracking the changes in spacing of densified gratings. This method has the potential to enable fabrication of integrated photonic system embedded within smart hydrogels.
